# Vulnerabilities of radiomic features to respiratory motion on four‐dimensional computed tomography‐based average intensity projection images: A phantom study

**DOI:** 10.1002/acm2.13498

**Published:** 2022-01-28

**Authors:** Takanori Adachi, Ryoko Nagasawa, Mitsuhiro Nakamura, Ryo Kakino, Takashi Mizowaki

**Affiliations:** ^1^ Division of Medical Physics Department of Information Technology and Medical Engineering Human Health Sciences Graduate School of Medicine Kyoto University Sakyo‐ku Japan; ^2^ Department of Radiation Oncology and Image‐Applied Therapy Graduate School of Medicine Kyoto University Sakyo‐ku Japan

**Keywords:** 4DCT‐based AIP, lung cancer, phantom study, radiomics, respiratory motion, robustness assessment

## Abstract

**Purpose:**

To evaluate the influence of respiratory motion on the robustness of radiomic features on four‐dimensional computed tomography (4DCT)‐based average intensity projection (AIP) images by employing an anthropomorphic chest phantom.

**Methods:**

Three spherical objects (φ30 mm), namely, acrylic (100 Hounsfield unit [HU], homogeneous), rubber (−140 HU, homogeneous), and cork (−630 HU, heterogeneous), were moved with motion amplitudes of 0, 1, 2.5, 4, 6, 8, and 10 mm in the phantom, and 4DCT scans were repeated at four different locations. Thereafter, the AIP images were generated considering the average of the 10 respiratory phases of the 4DCT images. Further, the targets were manually delineated on the AIP images in the lung window setting. A total of 851 radiomic features, including 107 unfiltered features and 744 wavelet filter‐based features, were extracted from the region of interest for each material. The feature robustness among the different target motion amplitude (*ε*) was evaluated by normalizing the feature variability of the target motion relative to the variability of data from 573 patients with early‐stage non‐small cell lung cancer. The features with absolute *ε* values ≤0.5 were considered highly robust to target motions.

**Results:**

The percentage of robust unfiltered and wavelet filter‐based features with a motion amplitude of 1 mm was greater than 83.2% and 93.4%, respectively; however, the percentage decreased by more than 24.3% and 17.6%, respectively, for motion amplitudes greater than 2.5 mm. The movement of cork had a small effect on the feature robustness compared to that of acrylic and rubber, regardless of the target motion amplitudes.

**Conclusions:**

Our phantom study demonstrated that target motion amplitudes ≤1 mm led to the robustness of radiomic features on the 4DCT‐based AIP images of thoracic regions. The frequency components and directions of the wavelet filters may be essential factors in 4DCT‐based radiomic analysis.

## 1 INTRODUCTION

Radiotherapy plays a crucial role in all stages of non‐small cell lung cancer (NSCLC).^1^ Stereotactic body radiation therapy (SBRT) is an effective therapeutic method for early‐stage NSCLC patients with inoperable tumors or those refusing surgical resection.^2^ Although lung SBRT substantially improves clinical outcomes compared with conventional fractionated radiotherapy, certain patients may develop local recurrence and distant metastasis following the treatment.^3^ Several studies have reported that many clinical factors (e.g., tumor size, histological type, and smoking history) influence the post‐SBRT prognosis; however, the prediction accuracy remains limited.^4^, ^5^

Recently, radiomic analysis has attracted considerable research attention owing to the extraction and analysis of multiple quantitative features as noninvasive biomarkers from medical images. For lung cancer, planning computed tomography (CT)‐based radiomic features are typically used to predict post‐SBRT prognosis.^6^, ^7^, ^8^ Although radiomic features may predict prognosis, the lack of generalization and stability is a challenge when performing radiomic analysis. Several studies have reported that the robustness of radiomic features depends on acquisition parameters such as slice thickness, scanner signal‐to‐noise ratio, and CT image reconstruction algorithm.^9^, ^10^ In addition to the above variabilities, respiratory‐induced tumor motion is considered as one of the largest uncertainties in lung cancer radiotherapy.^11^ Liu et al. demonstrated that the largest tumor motions in lung SBRT were observed in the superior–inferior (SI) direction, specifically, in the lower lung lobe.^12^ Moreover, they reported that 39.2% of lung tumors moved greater than 5 mm, while 10.8% of them moved greater than 10 mm. Four‐dimensional CT (4DCT) images are widely used in treatment planning to precisely capture these respiratory‐induced motions and accurately delineate patient‐specific internal gross tumor volumes.^13^, ^14^, ^15^ Larue et al. showed that 4DCT‐based phase images could be used to assess the radiomic feature robustness in the thoracic region.^16^ Further, Li et al. reported that respiratory motion‐related factors such as the respiratory amplitude, frequency, and 4DCT pitch exerted significant effects on the robustness of radiomic features from average intensity projection (AIP) images.^17^ Moreover, Du et al. investigated the stability of radiomic features across eight individual respiratory phases.^18^ To date, most 4DCT‐based radiomic analyses for the evaluation of feature robustness have been performed using patient data.^16^, ^17^, ^18^ However, no previous study has investigated the effect of respiratory amplitude on the robustness of radiomic features from 4DCT‐based AIP images in humans. As respiratory‐induced tumor motion cannot be accurately assessed owing to the large patient‐specific effects, a phantom study using an anthropomorphic chest phantom with a moving target was conducted to address this issue.

The purpose of this study was to evaluate the effects of target motion on the robustness of unfiltered and wavelet filters‐based radiomic features on 4DCT‐based AIP images using an anthropomorphic chest phantom. Inter‐target motion analysis was performed using three different homogeneous or heterogeneous materials with several target motion amplitudes.

## 2 MATERIALS AND METHODS

### 2.1 Phantom and experimental design

**FIGURE 1:**
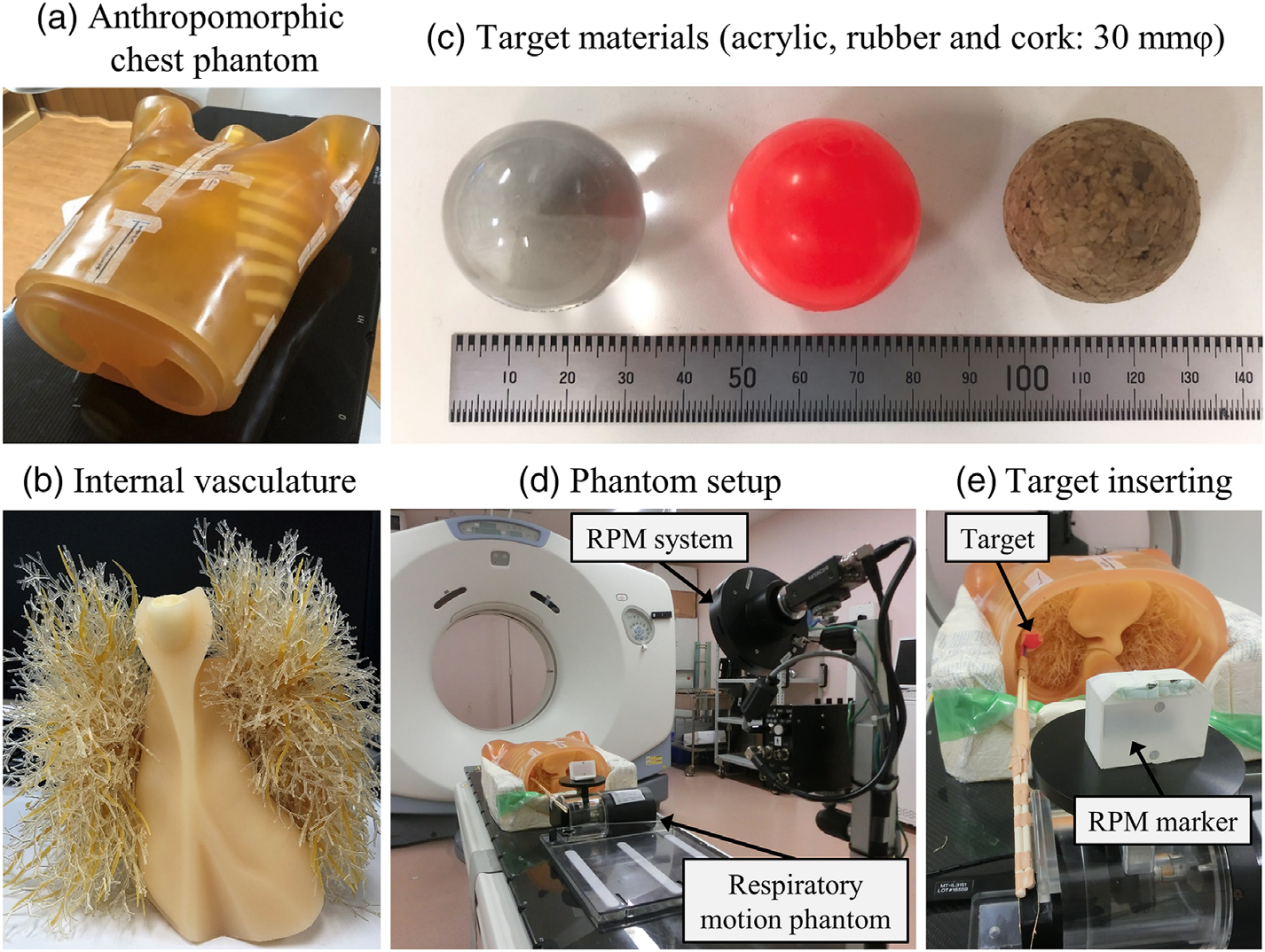
Chest phantom and experimental setup. (a) Anthropomorphic chest phantom body, (b) internal vasculature, (c) three spherical objects (φ30 mm): acrylic, rubber, and cork, (d) phantom and real‐time position management (RPM) system setup architecture, (e) target material insertion and RPM marker design

Figure 1 illustrates an anthropomorphic chest phantom and the experimental design. An anthropomorphic chest phantom (Kyoto Kagaku Co., Ltd, Kyoto, Japan) was used to simulate the human thoracic region (Figure 1a). The phantom consisted of a body structure and internal vasculature (Figure 1b). In the current study, three spherical objects (φ30 mm), namely, acrylic, rubber, and cork, were analyzed as target‐simulated materials (Figure 1c). Each material has different CT values (Hounsfield unit [HU]) and texture characteristics: acrylic (100 HU, homogeneous), rubber (−140 HU, homogeneous), and cork (−630 HU, heterogeneous).

The three target materials were connected to a QUASAR programmable respiratory motion phantom (Modus Medical Devices Inc., London, ON, Canada) and made to move in the SI direction (Figure 1d,e). The respiratory signal of the moving targets was recorded using a real‐time positioning management system for respiratory gating in the axial cine mode (version 1.7; Varian Medical Systems, Palo Alto, CA, USA) during the 4DCT scanning.^19^ Moreover, to simulate the different respiratory amplitudes, we analyzed seven motion amplitudes of 0, 1, 2.5, 4, 6, 8, and 10 mm (i.e., target motion ranges of 0, 2, 5, 8, 12, 16, and 20 mm) for each target material. The target position can be expressed as

(1) $$y\left(t\right)=A\sin\left(\frac{2\pi t}{T}-C\right)\;,$$

where *y*(*t*) denotes the target position at time *t*, *A* denotes the target motion amplitude, *T* denotes the motion period of 4 s, and *C* denotes the constant used to determine the starting phase of the target motion.

### 2.2 4DCT data acquisition and image reconstruction process

The 4DCT images were obtained in cine mode using a CT scanner system (LightSpeed RT16 11BW 46.3; General Electric Medical Systems, Waukesha, WI, USA). These image scans were repeated for each target material and motion amplitude at four different locations (upper central, upper peripheral, lower central, and lower peripheral) in the anthropomorphic chest phantom. The 4DCT scan parameters were as follows: tube voltage, 120 kV; tube current, 100 mA; gantry rotation time, 1.0 s/rot; scan duration, 6.0 s; and interscan delay, 1.3 s. Thereafter, the 4DCT images obtained were reconstructed employing a filtered back projection algorithm, with a slice thickness and field of view of 2.5 mm and 500 mm, respectively. Further, the 4DCT images and target motion data were transferred to an Advantage 4D Workstation (AW 4.5; General Electric Medical Systems), and AIP images were generated considering an average of 10 respiratory cycle phases of the 4DCT images. Subsequently, the AIP images were transferred to an Eclipse radiation treatment planning system (RTPS) (version 13.7.14; Varian Medical Systems) in the form of digital imaging and communications in medicine (DICOM) files. The data that support the findings of this study will be available from the corresponding author upon reasonable request.

### 2.3 Feature extraction

The target materials were manually delineated on the AIP images using Eclipse RTPS in the lung window setting (window width 1,500 HU; window level −600 HU). The contour information and CT image data were exported as DICOM files, which were subsequently converted to NRRD files using 3D slicer (version 4.10.2), an open‐source image processing and visualization system.^20^ For each scan data, a total of 851 CT‐based radiomic features were extracted from the region of interest (ROI) using a feature extraction software, Pyradiomics (version 2.2.0), with a resampled voxel size of 1 × 1 × 1 mm and a bin width of 25 HU.^21^ These features were defined with feature definitions as described by the Imaging Biomarker Standardization Initiative^22^ (Table S1). The texture features were derived from the gray‐level co‐occurrence matrix (GLCM), gray‐level dependence matrix (GLDM), gray‐level run‐length matrix, gray‐level size‐zone matrix, and neighboring gray‐tone difference matrix. Further, during the first order and texture feature extraction, wavelet filters, which decompose CT images into high‐ and low‐frequency components in the x (left–right), y (anterior–posterior), and z (SI) directions, were applied to extract multidimensional features. We analyzed 107 features (14 shape, 18 first order, and 75 texture features) without preprocessing and 744 features (144 first order and 600 texture features) with wavelet filters to 8 decompositions (i.e., LLL, LLH, LHL, LHH, HLL, HLH, HHL, and HHH).

### 2.4 Evaluation of the effect of target motion on radiomic features

**FIGURE 2:**
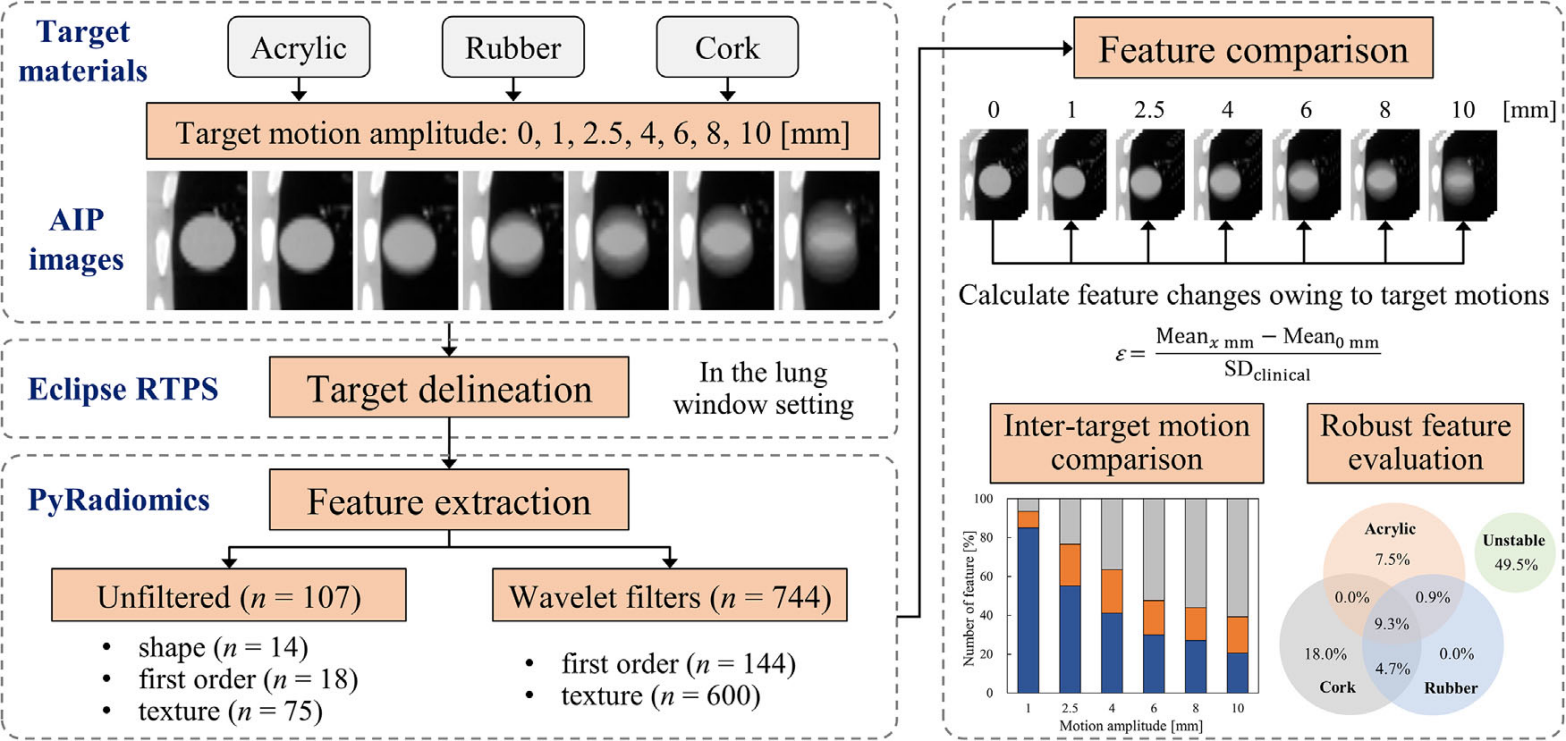
Workflow of the radiomic evaluation in this study. AIP, average intensity projection; Mean*_x_*_mm_, average of feature values extracted from four computed tomography images for target motion amplitude of *x* mm; Mean_0 mm_, average of feature values extracted from four computed tomography images for nontarget motion; RTPS, radiation treatment planning system; SD_clinical_, standard deviation of each feature value extracted from the clinical data

To relate the radiomic phantom studies to clinical applications, previous studies have converted different feature scales to the same scale in phantom data using the feature variability in patient data.^23^, ^24^ Thus, the current study used data from 573 early‐stage NSCLC patients who previously underwent SBRT at 11 institutions (Kyoto University Hospital and other institutions).^25^ The DICOM data consist of CT images with various scanner settings (i.e., 120 to 140 kV with tube currents controlled through the automatic exposure control technique) and contouring information as the structure sets. In the data of 573 patients, radiomic features were extracted from the gross tumor volume with the same parameter settings as in the current phantom study (i.e., resampled voxel size of 1 × 1 × 1 mm and bin width of 25 HU). To evaluate the effect of target motion on feature robustness (inter‐target motion comparison), we introduced an index, which is calculated as follows:

(2) $$\varepsilon =\frac{\mathrm{Mea}n_{x_{\mathrm{mm}}}-\mathrm{Mea}n_{0_{\mathrm{mm}}}}{SD_{\mathrm{clinical}}},$$

where Mean*_x_*_mm_ denotes the average of feature values extracted from four CT images for target motion amplitude of *x* mm, Mean_0 mm_ denotes the average of feature values extracted from four CT images for nontarget motion, and SD_clinical_ denotes the standard deviation (SD) of each feature value extracted from the clinical data.^25^ This normalization assessed the feature variability of the target motion relative to the variability of patient data. Because the CT parameters of the present phantom study were the most frequently used in the clinical study from 11 different institutions, the impact of differences in CT parameters on the robustness of the radiomic features would be small. Thereafter, referring to a previous study,^24^ all features were classified into the following: *ε* ≤ 0.5, high robustness; 0.5 < *ε* < 1.0, intermediate; and 1.0 ≤ *ε*, low robustness. The overall workflow of this study is shown in Figure 2.

## 3 RESULTS

### 3.1 Representative 4DCT‐based AIP images

**FIGURE 3:**
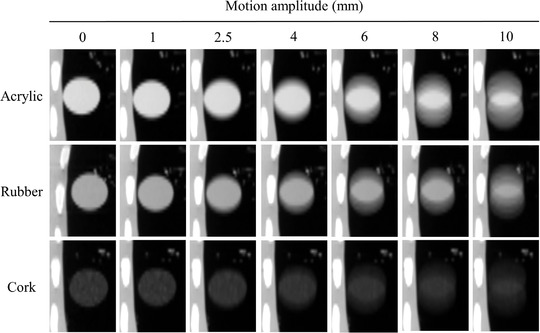
Representative images of three different materials, acrylic, rubber, and cork, at lower peripheral location in the anthropomorphic chest phantom. Target motion amplitude is 0, 1, 2.5, 4, 6, 8, and 10 mm for each material. Each image is displayed in a coronal plane at a window width of 1500 Hounsfield unit (HU) and window level of −600 HU. Each material has different computed tomography (CT) density and texture characteristics as follows: acrylic (100 HU, homogeneous), rubber (−140 HU, homogeneous), and cork (−630 HU, heterogeneous)

A total of 84 4DCT‐based AIP images (three materials × seven motion amplitudes × four experimental times) were acquired. Figure 3 shows representative AIP images of acrylic, rubber, and cork with different target motion amplitudes at the lower peripheral locations in the anthropomorphic chest phantom. The motion of each target material in the SI direction was visually confirmed on the AIP image.

### 3.2 Radiomic feature robustness for target motion amplitudes

Before assessing the impact of target motion on radiomic features, we evaluated the repeatability of the phantom experiment by performing the same experiment twice without the target movement. The number of highly repeatable features was 92.5%–100.0%, 86.0%–100.0%, and 86.9%–100.0% in the acrylic, rubber, and cork, respectively (Figure S1).

**FIGURE 4:**
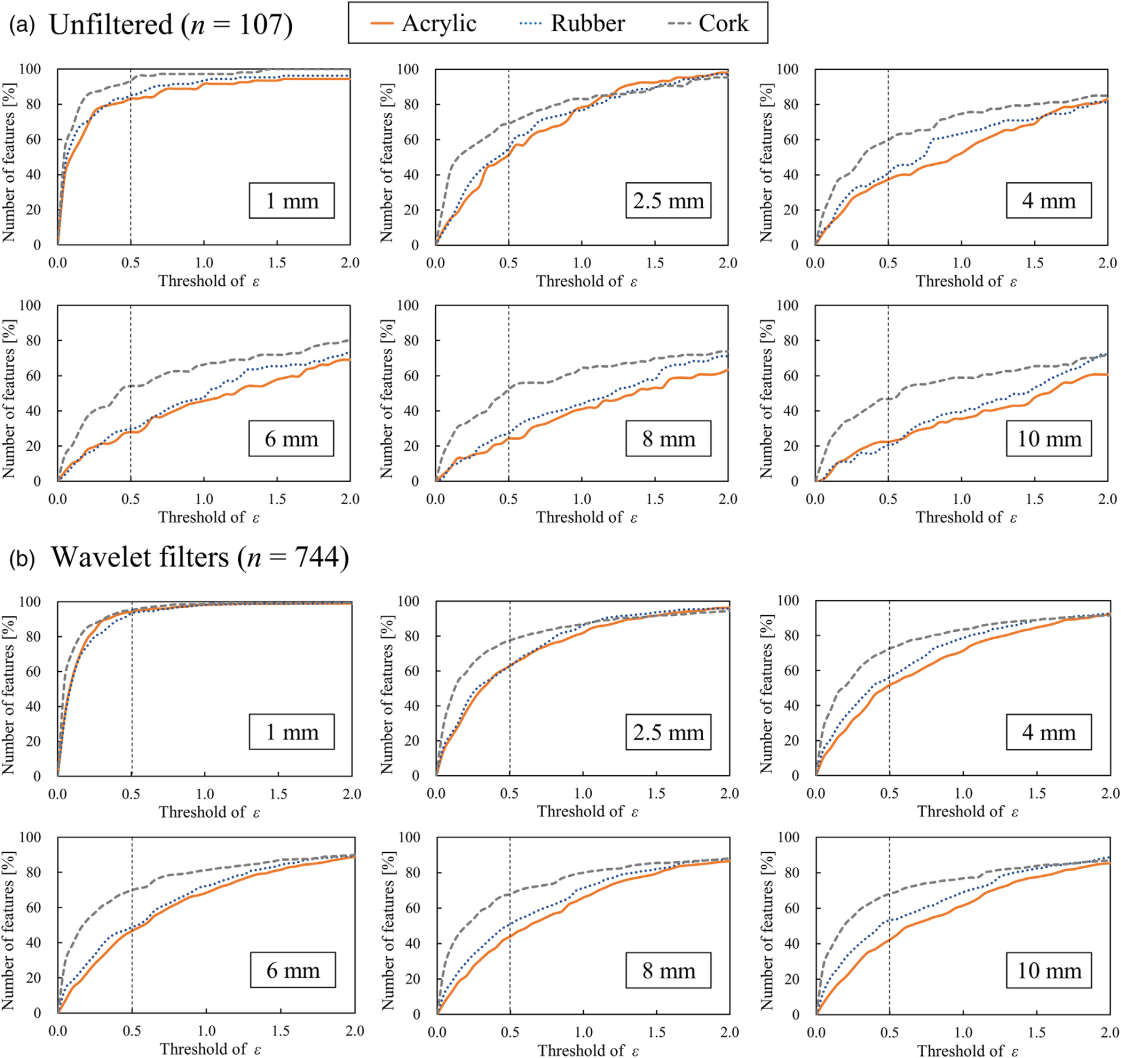
Percentage of highly robust (a) unfiltered and (b) wavelet filter‐based features regarding target motion amplitudes of 1, 2.5, 4, 6, 8, and 10 mm for different thresholds of *ε* value for the acrylic, rubber, and cork. Vertical axis shows the number of highly robust features; horizontal axis shows different thresholds of *ε* value

Figure 4 shows the percentage of highly robust features regarding the target motion amplitudes for each material for different *ε* value thresholds. The percentage of robust unfiltered and wavelet filter‐based features with motion amplitudes of 1 mm for acrylic, rubber, and cork were determined to be 83.2 and 94.2, 85.0 and 93.4, and 93.5 and 95.2%, respectively. However, for motion amplitudes greater than 2.5 mm, these percentages for acrylic, rubber, and cork decreased by more than 31.8% and 31.5%, 29.9% and 30.8%, and 24.3% and 17.6%, respectively. It was observed that the robust features of the cork were greater than those of the acrylic and rubber at any *ε* value threshold, regardless of the target motion amplitudes.

### 3.3 Effects of wavelet filters on the robustness of radiomic features

**FIGURE 5:**
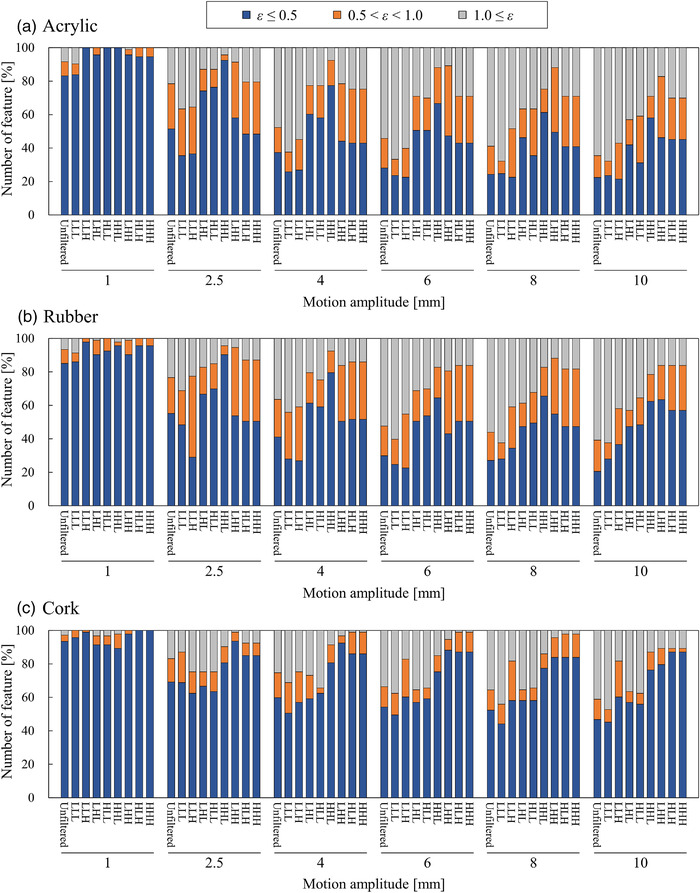
Proportion of radiomic features based on *ε* value to evaluate target motion amplitudes for (a) acrylic, (b) rubber, and (c) cork. Radiomic features were classified into the following: *ε* ≤ 0.5; high robustness; 0.5 < *ε* < 1.0; intermediate; 1.0 ≤ *ε*; low robustness. Wavelet filters were used in eight frequency decompositions—LLL, LLH, LHL, HLL, LHH, HLH, HHL, and HHH. Vertical axis shows the number of highly robust features; horizontal axis shows different target motion amplitudes of 1, 2.5, 4, 6, 8, and 10 mm

Figure 5 depicts the proportion of radiomic features based on the *ε* value that evaluates target motion amplitudes for (a) acrylic, (b) rubber, and (c) cork. For the acrylic and rubber, the wavelet filter‐based features with LHL/HLL/HHL and LHH/HLH/HHH decompositions improved the robustness to target motion compared to unfiltered features. Specifically, the percentage of robust wavelet filter‐based features with LHL/HLL/HHL decompositions for acrylic and rubber at motion amplitudes of 2.5, 4, and 6 mm increased by more than 22.8, 20.7, and 22.5% and 11.5, 18.0, and 20.6%, respectively. Further, when the motion amplitudes of the acrylic and rubber were greater than 2.5 mm, the effects of different motion amplitudes on the robustness of wavelet filter‐based features with LHH/HLH/HHH decompositions were small, that is, in the range of 40.9%–58.1% and 43.0%–63.4%, respectively. By contrast, when the motion amplitude of the cork was greater than 2.5 mm, the percentage of robust wavelet filter‐based features with LHH/HLH/HHH decompositions was increased by more than 15.8%, compared to unfiltered features.

### 3.4 Overlapped robust radiomic features

**FIGURE 6:**
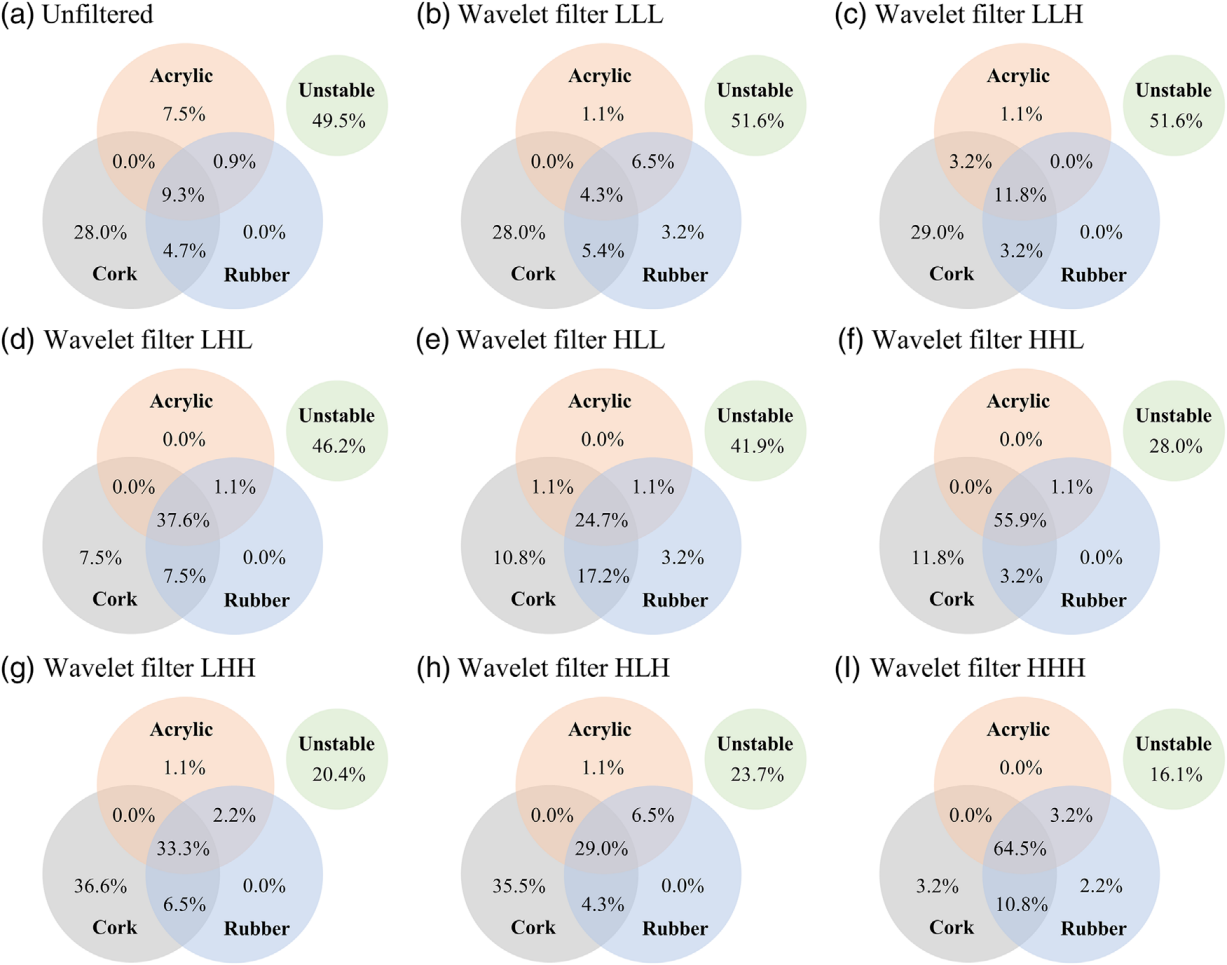
Venn diagram visualizing the overlap of highly robust (a) unfiltered features and wavelet filter‐based features with (b) LLL, (c) LLH, (d) LHL, (e) HLL, (f) HHL, (g) LHH, (h) HLH, and (i) HHH regarding the acrylic, rubber, and cork for target motion amplitudes of 1, 2.5, 4, 6, 8, and 10 mm

Figure 6 shows a Venn diagram that visualizes the overlap of highly robust features regarding the acrylic, rubber, and cork for all motion amplitudes. Compared to the unfiltered features, the percentage of robust wavelet filter‐based features with LHL/HLL/HHL and LHH/HLH/HHH decompositions increased by more than 15.4% and 19.7%, respectively. Table 1 summarizes the overlapped radiomic feature groups considering the three target materials for all motion amplitudes. In the case of wavelet filters with HHL and HHH decompositions, the most robust feature group was the first order, followed by the GLCM and GLDM feature groups.

**TABLE 1:** Overlapped radiomic feature groups regarding target materials for all motion amplitudes

Feature groups (number of features)		Wavelet filters							
	Unfiltered	LLL	LLH	LHL	HLL	HHL	LHH	HLH	HHH
Shape (14)	5 (35.7%)	–	–	–	–	–	–	–	–
First order (18)	2 (11.1%)	2 (11.1%)	1 (5.6%)	5 (27.8%)	3 (16.7%)	13 (72.2%)	5 (27.8%)	4 (22.2%)	15 (83.3%)
GLCM (24)	0 (0.0%)	0 (0.0%)	2 (8.3%)	9 (37.5%)	6 (25.0%)	16 (66.7%)	9 (37.5%)	7 (29.2%)	18 (75.0%)
GLDM (14)	0 (0.0%)	0 (0.0%)	2 (12.5%)	5 (31.3%)	3 (18.8%)	7 (43.8%)	4 (25.0%)	3 (18.8%)	11 (68.8%)
GLRLM (16)	0 (0.0%)	0 (0.0%)	2 (12.5%)	5 (31.3%)	4 (25.0%)	6 (37.5%)	5 (31.3%)	5 (31.3%)	6 (37.5%)
GLSZM (16)	1 (7.1%)	0 (0.0%)	2 (14.3%)	7 (50.0%)	3 (21.4%)	6 (42.9%)	5 (35.7%)	5 (35.7%)	7 (50.0%)
NGTDM (5)	2 (40.0%)	2 (40.0%)	2 (40.0%)	4 (80.0%)	4 (80.0%)	4 (80.0%)	3 (60.0%)	3 (60.0%)	3 (60.0%)

Note: Each value represents the number of robust features (percentage) of the target materials.

Abbreviations: GLCM, gray‐level co‐occurrence matrix; GLDM, gray‐level dependence matrix; GLRLM, gray‐level run‐length matrix; GLSZM, gray‐level size‐zone matrix; NGTDM, neighboring gray‐tone difference matrix.

## 4 DISCUSSION

Respiratory motion is a major uncertainty in lung cancer radiotherapy. Thus, it is important to assess the uncertainty caused by respiratory tumor motion in radiomic analysis for prognostic prediction after lung cancer radiotherapy. To the best of our knowledge, no previous studies have evaluated the effect of various target movements on the robustness of features using several target materials with different CT values and homogeneity. In this study, we observed that a target motion amplitude greater than 2.5 mm significantly affected the robustness of radiomic features on 4DCT‐based AIP images regardless of the target materials. Further, we found that for target motion amplitudes of 2.5, 4, and 6 mm, low‐frequency decomposition of wavelet filters (i.e., wavelet filters with LHL/HLL/HHL decompositions) in the SI direction improved the robustness of radiomic features.

Our results indicated that the number of robust features decreased as the target motion increased. The radiomic features were calculated based on the CT values of each voxel within the ROIs. Further, as the AIP images used in the current study were obtained by averaging 10 respiratory phases of 4DCT images, an increase in the target motion resulted in a decrease in the brightness because of the low CT value (lung fields) inside the ROIs. In addition, cork (heterogeneous material) exhibited greater robustness compared to that of acrylic and rubber (homogenous materials). This is because when the heterogeneous material has no movement, there is a contrast in the CT value of each voxel inside the target. Conversely, when the homogenous material does not move, the CT value is approximately uniform, resulting in a small contrast inside the target. Hence, when the robustness of the feature is calculated based on the case of no movement, it can be considered that the robustness of the homogeneous material decreased owing to the significant effect of the variation in CT value. Moreover, the effects of the CT value on the feature robustness were small compared to the homogenous materials of acrylic (100 HU) and rubber (−140 HU). The acrylic with a higher CT value had greater noise in the voxel compared to the rubber. However, these effects may be minimal when lung regions with extremely low CT values are included owing to the target movement. Therefore, the heterogeneity of the material affects the robustness of radiomic features on 4DCT‐based AIP images regarding target movements compared to CT values.

Considering the current clinical situation, the effective management of respiratory motion is essential for achieving clinical goals. However, certain concerns regarding the techniques used to assess the effects of tumor motion on the robustness of radiomic features exist. Several studies have reported that the gated or breath‐hold CT approach is required to minimize the impact of tumor motion to enable the selection of generalizable and robust radiomic features.^26^, ^27^ However, clinical protocols for CT imaging may not be standardized in each institution, and it may be difficult to acquire gated or breath‐hold CT owing to technical problems or poor patient conditions. In this study, different target motion amplitudes were employed to investigate the precise target motion effects on the robustness of radiomic features. As shown in Figure 5, the feature robustness decreased as the target motion amplitudes increased from 2.5 to 10 mm. Therefore, the results suggest that the robustness of radiomic features on 4DCT‐based AIP images can be improved in 4DCT by suppressing the target movement as much as possible. Thus, the suppression of respiratory motion may be essential for the robustness of radiomic analysis of 4DCT‐based AIP images.

The application of the wavelet filters is one of the important factors that affects the robustness of radiomic features. Larue et al. investigated the stability of radiomic features on 4DCT images (from eight breathing phases) in the thoracic region and found that the stability of unfiltered features was higher than that of wavelet filter‐based features.^16^ Although they applied wavelet filters to their radiomic analysis, the frequency components or direction of wavelet filters was not considered. Our findings indicated that applying low‐frequency decompositions of wavelet filters (i.e., wavelet filters with LHL/HLL/HHL decompositions) in the SI direction improved the robustness of radiomic features for the target motion amplitudes of 2.5–6 mm (Figure 5). This is because the wavelet filters with low‐frequency components smoothed out the blurring caused by the target movement on AIP images. By contrast, for motion amplitudes of 2.5–10 mm, the robustness of wavelet filters with LHH/HLH/HHH decompositions was almost the same because it enhanced the blurring caused by target movement on the AIP images. Therefore, the frequency components and directions of the wavelet filters are the essential factors in radiomic‐based prognostic prediction for thoracic regions on AIP images.

For radiomic analysis in lung SBRT, Huynh et al. reported that AIP images outperformed free‐breathing (FB) images for the prediction of distant failure following SBRT because AIP images contain greater prognostic information than FB images.^27^ The radiomic features on 4DCT‐based AIP images express the respiratory movement of the target and contain various respiratory phase information; thus, the AIP images may be effective for radiomics‐based prognosis prediction. By contrast, Davey et al. investigated the most stable 4DCT phase for radiomic analysis^28^ and reported that although the most stable phase varies among individual patients, the selection of radiomic features for predicting distant failure improved the model performance compared to the use of nonselective methods. To rephrase, in the radiomic analysis of lung tumors with respiratory movement, it may be appropriate to use 4DCT images that include the information of respiratory tumor movement. Furthermore, in the prognosis prediction by radiomic analysis based on 4DCT images with various respiratory phases, the details of the feature extracted images and the robustness assessment scenario of the radiomic analysis must be reported.

However, several limitations exist in the present study, which warrant further discussion. First, because the target motion was simulated only in the SI direction, the direct clinical implementation is limited. Each patient had an irregular respiratory movement pattern in the three‐dimensional directions, such as various motion amplitudes and respiratory cycles during imaging, which may affect the blurring of 4DCT‐based AIP images. However, the tumor predominantly moves in the SI direction,^12^, ^17^ and previous phantom study simulated the tumor movement predominantly in the SI direction.^17^ Further studies are required to identify the effects of precise target motions in three‐dimensional directions on radiomic feature reproducibility. Another limitation is the feature evaluation index used for evaluating the radiomic feature robustness owing to the small sample sizes of the phantom experiments. Several studies have assessed radiomic robustness using the concordance correlation coefficient (CCC) or coefficient of variation (COV).^10^, ^16^, ^17^, ^18^ However, the CCC attains an extremely small value when the sample size is limited. In addition, although the COV can be used to evaluate feature variability regardless of the feature scale using the mean and SD of each feature, it is not appropriate for the current study because the SD differs for each material. It is desirable to compare the results of this study with those of other studies directly using CCC and COV. However, referring to a previous study,^24^ the feature evaluation index, which is derived from the SD of patient data, is appropriate for addressing both of the above issues in the current study. The third limitation is the slice thickness of the 4DCT images. In the present study, the motion amplitude of 1 mm had a small effect on the robustness of radiomic features, but this amplitude was smaller than the current slice thickness of 2.5 mm. It is unknown whether similar results regarding feature robustness would be obtained when the slice thickness is set to 1 mm. However, according to the report of the American Association of Physicists in Medicine Task Group 101, the recommended slice thickness for lung SBRT is 1–3 mm.^29^ Furthermore, because a slice thickness of 2–3 mm is most frequently used in Japan,^30^ the results of this study are clinically reasonable.

## 5 CONCLUSIONS

This phantom study investigated the effect of target motion of three different materials (acrylic, rubber, and cork) on the radiomic features extracted from 4DCT‐based AIP images. Our results demonstrated that a smaller target motion led to the robustness of unfiltered and wavelet filter‐based radiomic features, irrespective of the different textured materials. Notably, it was determined that the robustness of the cork (heterogeneous) to target movement was greater than that of acrylic and rubber (homogeneous). For acrylic and rubber, the radiomic features would be robust to target motion when applying wavelet filters with low‐frequency decomposition in the direction of target movement. In a clinical situation, the suppression of respiratory motion may support the development of a standardized radiomic approach for thoracic regions.

## CONFLICT OF INTEREST

The authors have no competing financial relationships to disclose.

## Supporting information

Supporting information

Supporting information
