# Supplementary figures and images for: Vulnerabilities of radiomic features to respiratory motion on four‐dimensional computed tomography‐based average intensity projection images: A phantom study
Source: J Appl Clin Med Phys. 2022 Jan 28;23(3):e13498. doi: 10.1002/acm2.13498 (PMC8906211; doi:10.1002/acm2.13498)

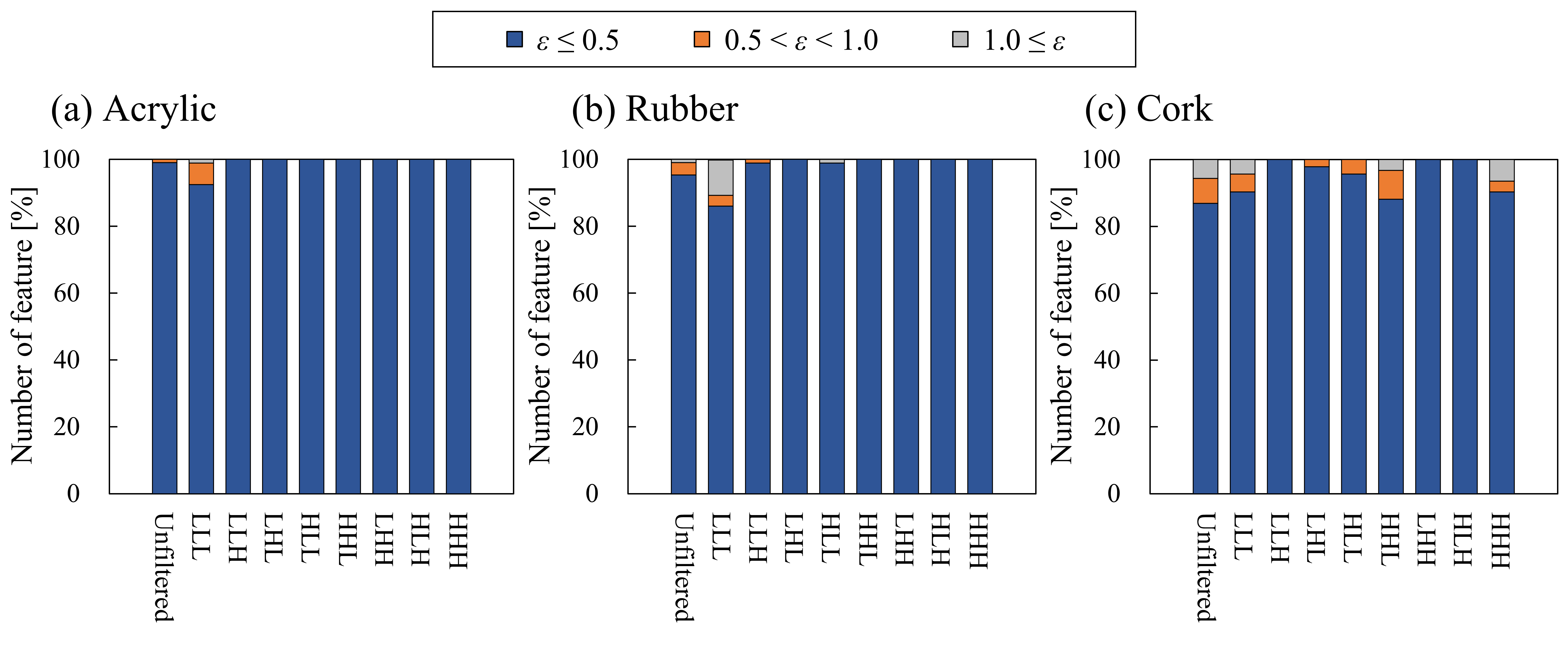

Supplement: Supplementary file 1 — Supporting information [file ACM2-23-e13498-s001.tif]
